# Supplementary material for: Temporal deposition of copper and zinc in the sediments of metal removal constructed wetlands
Source: PLoS One. 2021 Aug 3;16(8):e0255527. doi: 10.1371/journal.pone.0255527 (PMC8330884; doi:10.1371/journal.pone.0255527)
Supplement: S1 Fig — (DOCX) [file pone.0255527.s001.docx]

**
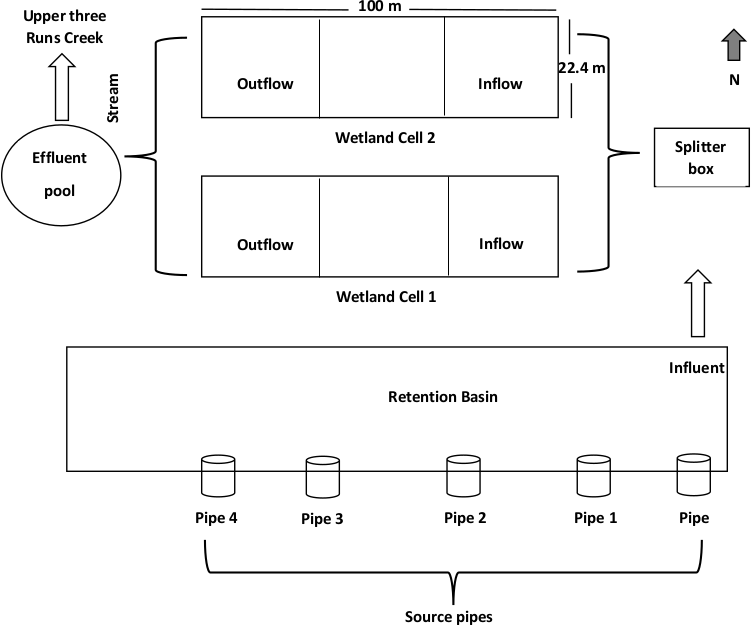
**

**Figure S1** Schematic diagram of the H-02 Wetland system showing source pipes, water flow, and sediment core sampling locations (Inflow/Outflow) in both wetland cells 1 and 2 (after[1])
