# Supplementary figures and images for: Temporal deposition of copper and zinc in the sediments of metal removal constructed wetlands
Source: PLoS One. 2021 Aug 3;16(8):e0255527. doi: 10.1371/journal.pone.0255527 (PMC8330884; doi:10.1371/journal.pone.0255527)

**
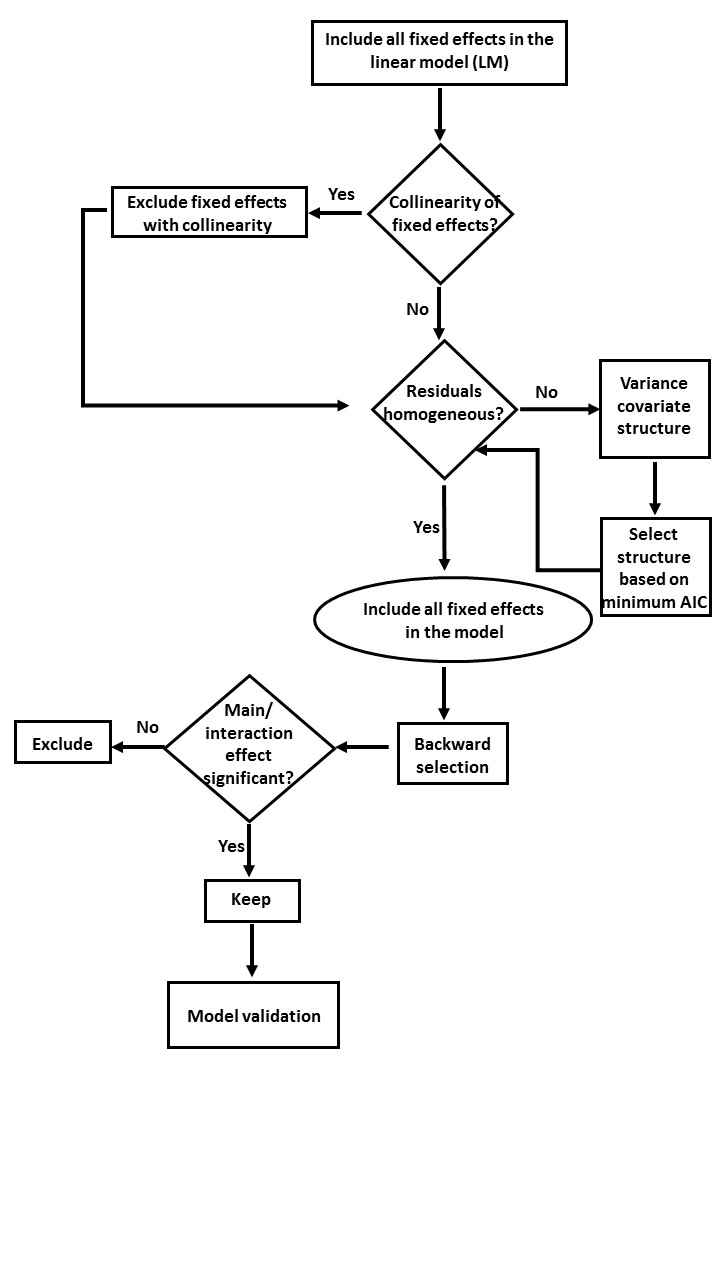
**

**Figure S2** Flowchart of the model selection process

Supplement: S2 Fig — (DOCX) [file pone.0255527.s002.docx]

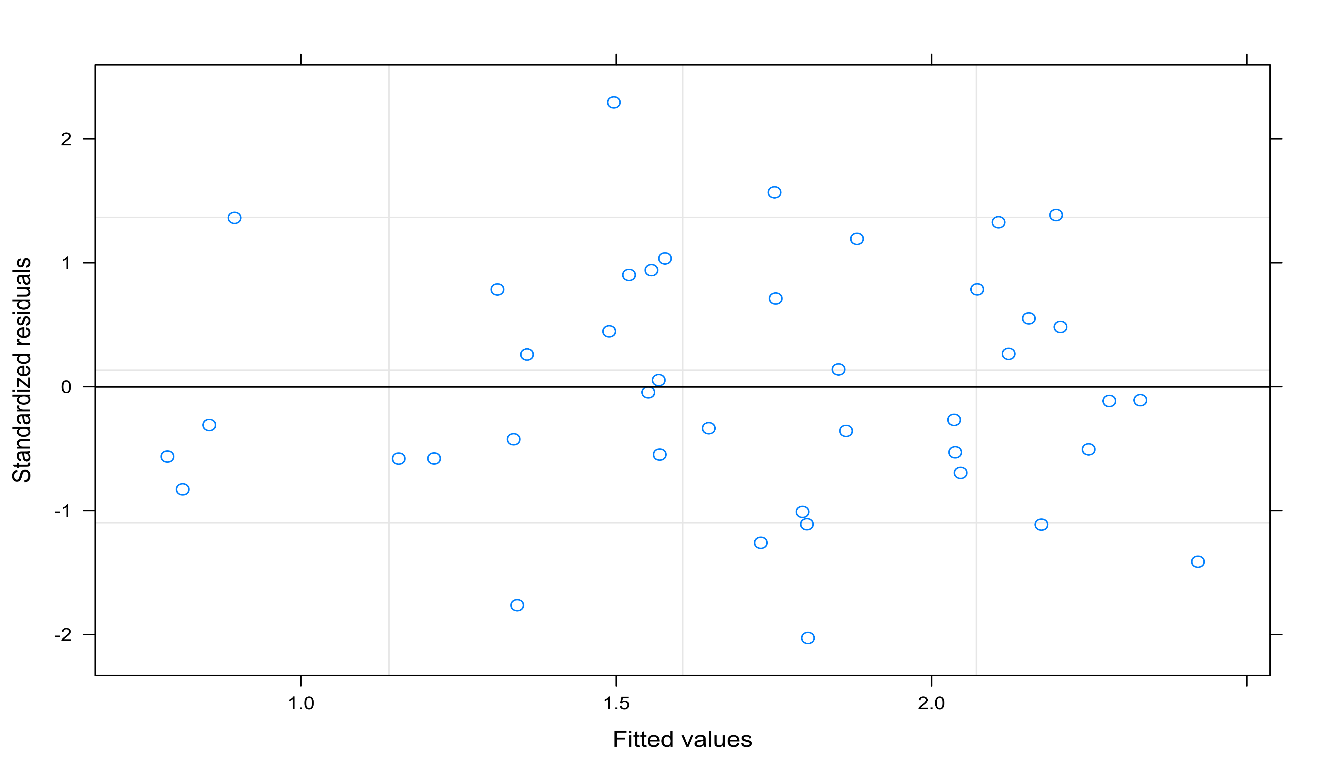


**Figure S4** Standardized residuals vs fitted values for the linear model (lm) for Cu

Supplement: S4 Fig — (DOCX) [file pone.0255527.s004.docx]

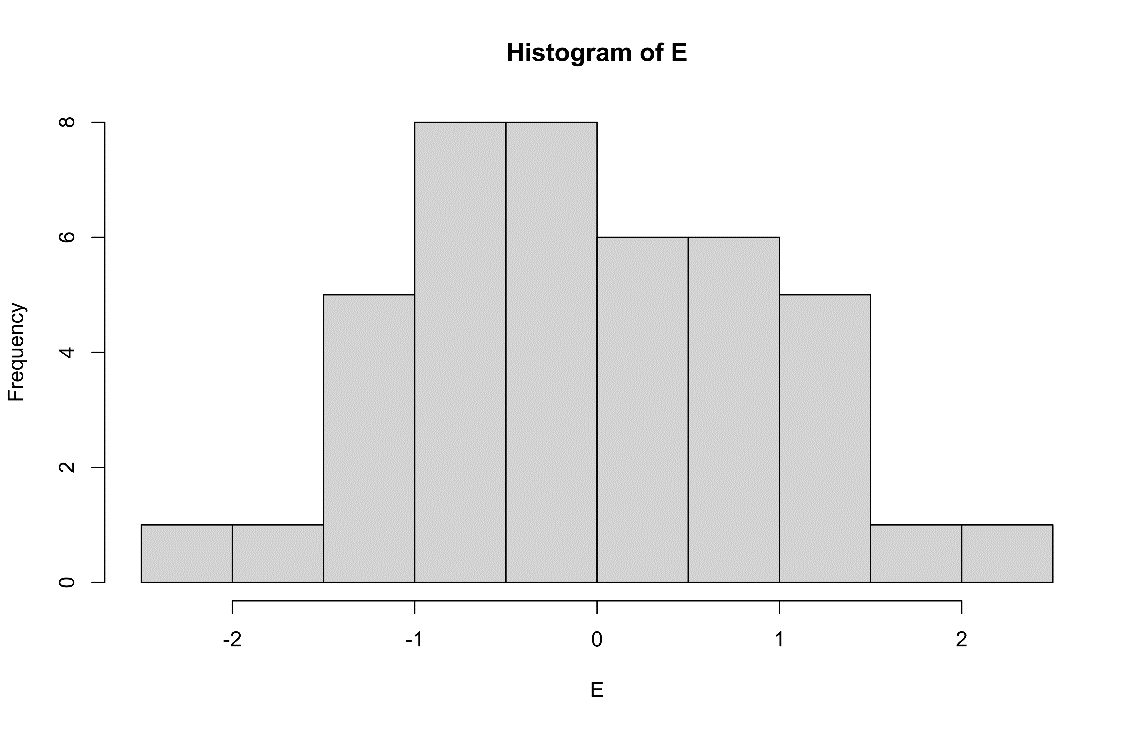


**Figure S5** Pearson’s normalized residuals denoted by E for the linear model (lm) for Cu

Supplement: S5 Fig — (DOCX) [file pone.0255527.s005.docx]
