# Supplementary material for: Temporal deposition of copper and zinc in the sediments of metal removal constructed wetlands
Source: PLoS One. 2021 Aug 3;16(8):e0255527. doi: 10.1371/journal.pone.0255527 (PMC8330884; doi:10.1371/journal.pone.0255527)
Supplement: S3 Fig — (DOCX) [file pone.0255527.s003.docx]

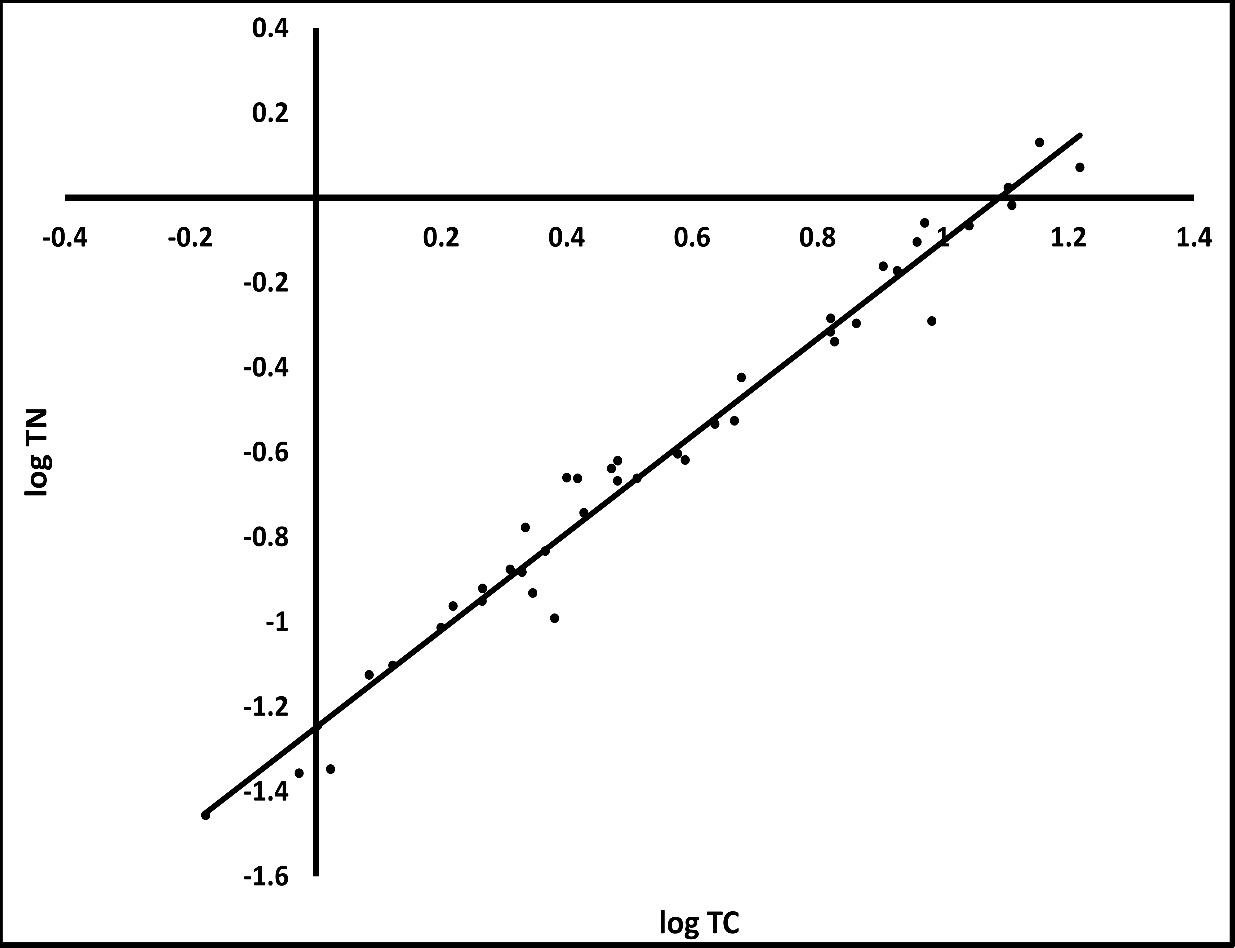


**Figure S3** Relationship between log transformed total carbon (log_10_TC) and total nitrogen (log_10_TN),

Correlation coefficient, R^2^ = 0.93.
