# Supplementary material for: Temporal deposition of copper and zinc in the sediments of metal removal constructed wetlands
Source: PLoS One. 2021 Aug 3;16(8):e0255527. doi: 10.1371/journal.pone.0255527 (PMC8330884; doi:10.1371/journal.pone.0255527)
Supplement: S6 Fig — (DOCX) [file pone.0255527.s006.docx]

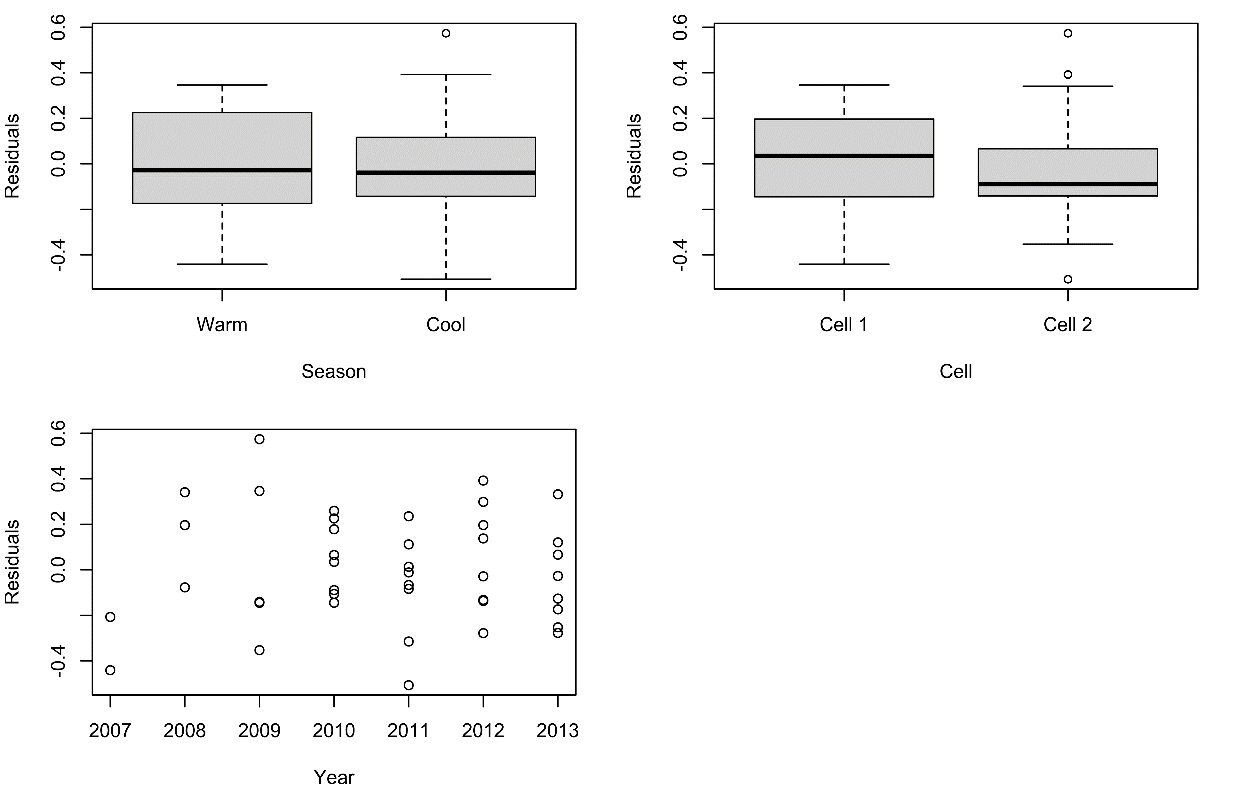


**Figure S6** Pearson’s normalized residuals for the linear model (lm) for Cu plotted against seasons (warm and cool), Cells (1 and 2), and the years of the study (2007-2013)
