# Supplementary material for: Temporal deposition of copper and zinc in the sediments of metal removal constructed wetlands
Source: PLoS One. 2021 Aug 3;16(8):e0255527. doi: 10.1371/journal.pone.0255527 (PMC8330884; doi:10.1371/journal.pone.0255527)
Supplement: S8 Fig — (DOCX) [file pone.0255527.s008.docx]

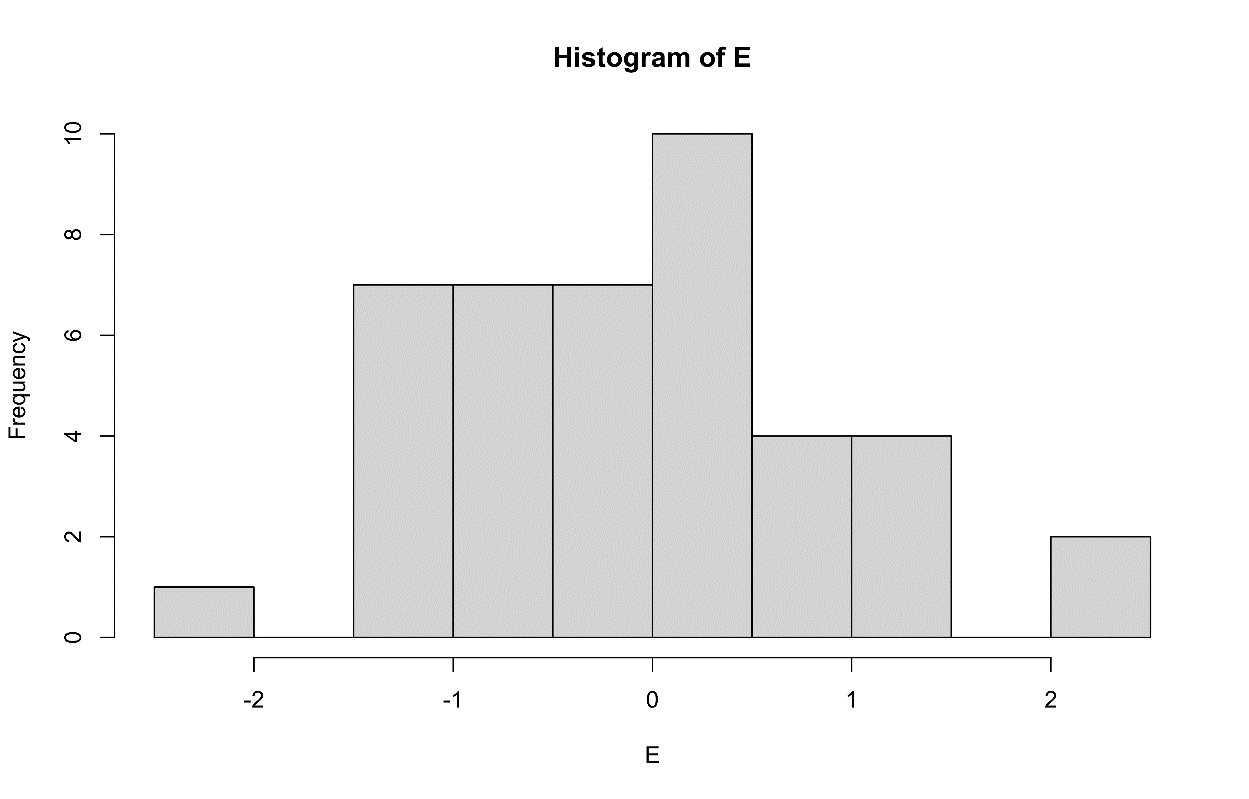


**Figure S8** Pearson’s normalized residuals denoted by E for the linear model with generalized least squares extension (gls) for Zn
