# Supplementary material for: Temporal deposition of copper and zinc in the sediments of metal removal constructed wetlands
Source: PLoS One. 2021 Aug 3;16(8):e0255527. doi: 10.1371/journal.pone.0255527 (PMC8330884; doi:10.1371/journal.pone.0255527)
Supplement: S9 Fig — (DOCX) [file pone.0255527.s009.docx]

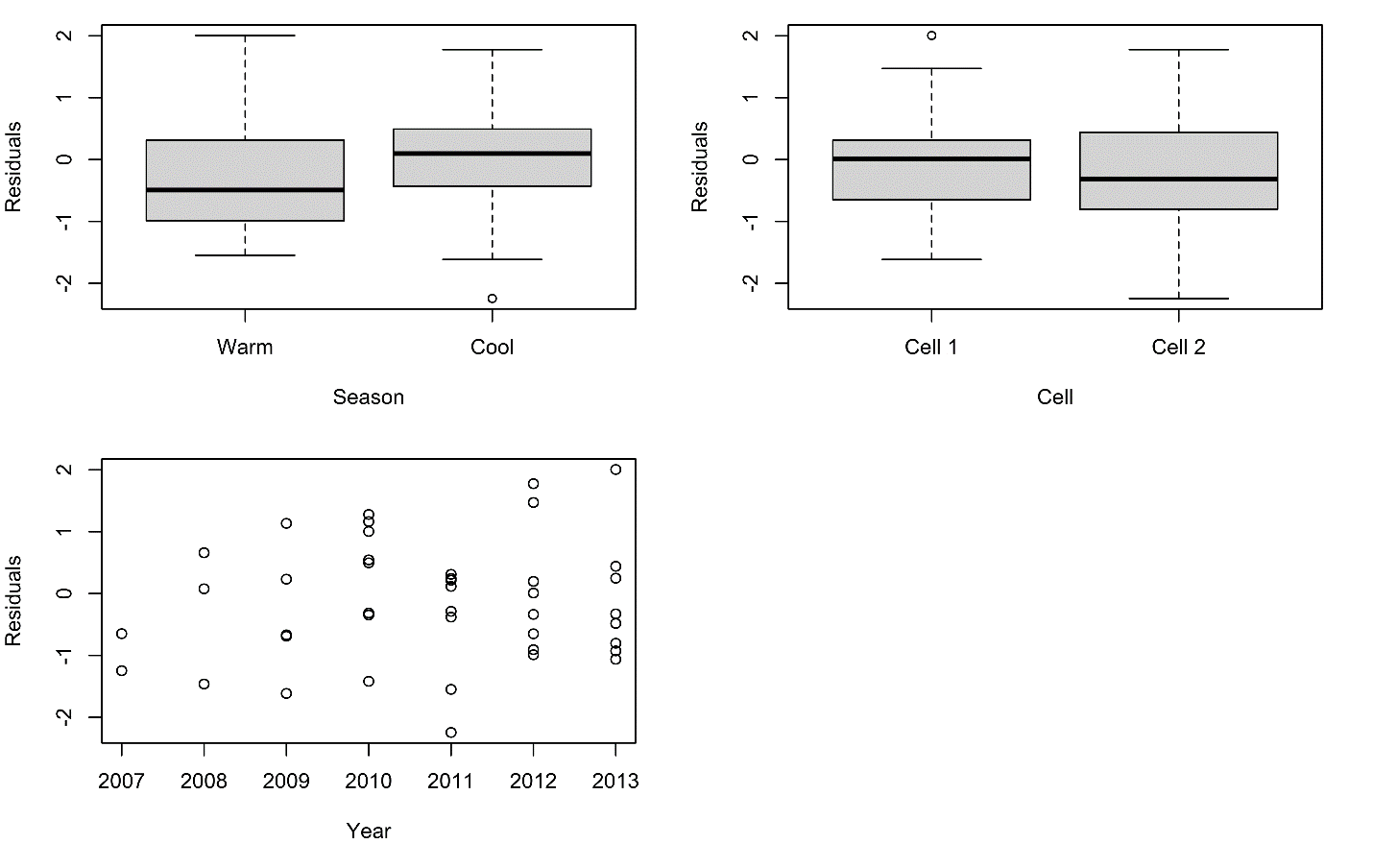


**Figure S9** Pearson’s normalized residuals for the linear model with generalized least squares extension (gls) for Zn plotted against seasons (warm and cool), Cells (1 and 2), and the years of the study (2007-2013)
