# Supplementary material for: Temporal deposition of copper and zinc in the sediments of metal removal constructed wetlands
Source: PLoS One. 2021 Aug 3;16(8):e0255527. doi: 10.1371/journal.pone.0255527 (PMC8330884; doi:10.1371/journal.pone.0255527)
Supplement: S10 Fig — (DOCX) [file pone.0255527.s010.docx]

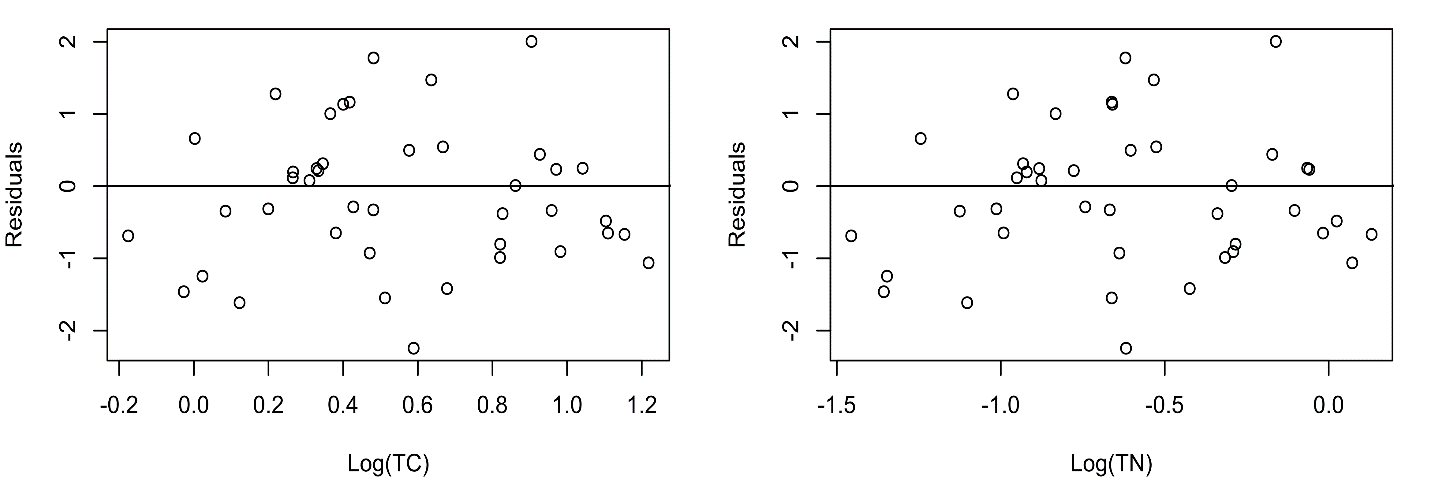


**Figure S10** Pearson’s normalized residuals of the linear model with generalized least squares extension (gls) for Zn plotted against log_10_TC and log_10_TN
