# Supplementary material for: Temporal deposition of copper and zinc in the sediments of metal removal constructed wetlands
Source: PLoS One. 2021 Aug 3;16(8):e0255527. doi: 10.1371/journal.pone.0255527 (PMC8330884; doi:10.1371/journal.pone.0255527)
Supplement: S1 Table — (DOCX) [file pone.0255527.s011.docx]

**Table S1** Variance inflation factors (VIF) of fixed effects included in the generalized linear model for Cu

| **Year** | **Season** | **Log_10_TC** | **Log_10_TN** | **Cell** |
| --- | --- | --- | --- | --- |
| 1.799926 | 1.110638 | 19.808843 | 17.197561 | 1.086630 |
