# Supplementary material for: Temporal deposition of copper and zinc in the sediments of metal removal constructed wetlands
Source: PLoS One. 2021 Aug 3;16(8):e0255527. doi: 10.1371/journal.pone.0255527 (PMC8330884; doi:10.1371/journal.pone.0255527)
Supplement: S2 Table — (DOCX) [file pone.0255527.s012.docx]

**Table S2** Concentration of Cu (mg kg^-1^ dry weight) in each sediment layer (Top, Middle, and Bottom) per year. Each concentration is represented by the mean ± 95% confidence intervals.

| **Year** | **Layer** | **By season** | | **By location** | | **By cell** | |
| --- | --- | --- | --- | --- | --- | --- | --- |
|  |  | **Warm** | **Cool** | **Inflow** | **Outflow** | **1** | **2** |
| 2007 | Top | 5.3±7.5 | NA | 4.6±47.1 | 6.01±50.2 | 7.2±19.4 | 3.4±16.4 |
|  | Middle | 5.9±3.5 | NA | 5.5±3.7 | 6.2±31.9 | 6.6±22.4 | 5.2±5.8 |
|  | Bottom | 5.9±0.9 | NA | 6.2±0.8 | 5.7±3.7 | 6.0±3.6 | 5.9±8.0 |
| 2008 | Top | 13.2±16.9 | 93.2±293.8 | 63.1±318.0 | 43.2±161.3 | 67.1±311.3 | 39.2±166.4 |
|  | Middle | 8.4±3.3 | 5.6±2.2 | 6.4±5.3 | 7.1±4.8 | 6.9±4.6 | 6.8±5.9 |
|  | Bottom | 6.5±3.6 | NA | 5.9±9.6 | 7.0±4.8 | 7.3±5.4 | 5.5±7.9 |
| 2009 | Top | 224.4±350.0 | 23.1±48.6 | 125.9±302.6 | 120.5±314.1 | 104.6±521.5 | 136.5±295.8 |
|  | Middle | 7.0±5.2 | 6.3±4.0 | 6.5±4.0 | 6.8±5.6 | 7.1±4.6 | 6.3±4.3 |
|  | Bottom | 6.5±2.0 | NA | 6.2±2.2 | 6.8±5.7 | 6.7±1.9 | 6.3±4.3 |
| 2010 | Top | 203.7±421.3 | 144.8±351.5 | 298.4±453.9 | 50.2±47.1 | 145.0±344.9 | 203.6±426.8 |
|  | Middle | 6.9±4.6 | 5.9±3.2 | 6.7±1.2 | 6.1±5.0 | 6.6±3.3 | 5.9±3.2 |
|  | Bottom | 6.6±2.4 | 6.0±4.1 | 5.7±3.0 | 7.1±2.6 | 6.4±2.0 | 6.4±3.8 |
| 2011 | Top | 151.9±258.3 | 103.2±200.7 | 211.3±253.9 | 43.7±39.1 | 140.6±204.3 | 114.4±260.8 |
|  | Middle | 7.9±7.0 | 8.0±5.8 | 8.1±6.9 | 7.8±6.0 | 7.8±7.1 | 8.1±5.7 |
|  | Bottom | 4.8±1.9 | 7.2±6.4 | 6.7±6.9 | 5.4±1.6 | 7.4±7.5 | 5.0±2.1 |
| 2012 | Top | 265.0±365.5 | 243.4±379.1 | 378.7±410.5 | 129.7±102.1 | 278.3±368.5 | 230.0±372.1 |
|  | Middle | 19.7±43.0 | 8.1±5.9 | 10.1±10.6 | 15.6±31.2 | 16.3±34.8 | 10.2±17.6 |
|  | Bottom | 4.4±1.6 | 5.4±3.3 | 5.1±3.8 | 4.8±1.0 | 5.4±2.3 | 4.4±3.1 |
| 2013 | Top | 146.9±123.6 | 240.7±293.0 | 236.7±249.2 | 139.6±146.6 | 215.1±217.7 | 169.6±228.4 |
|  | Middle | 6.9±3.3 | 14.4±25.9 | 16.2±30.4 | 6.3±2.0 | 10.9±18.2 | 12.1±41.6 |
|  | Bottom | NA | 6.1±3.9 | 5.8±7.1 | 6.5±25.5 | 6.9±7.5 | 5.2±10.0 |
