# Supplementary material for: Temporal deposition of copper and zinc in the sediments of metal removal constructed wetlands
Source: PLoS One. 2021 Aug 3;16(8):e0255527. doi: 10.1371/journal.pone.0255527 (PMC8330884; doi:10.1371/journal.pone.0255527)
Supplement: S3 Table — (DOCX) [file pone.0255527.s013.docx]

**Table S3** Concentration of Zn (mg kg^-1^ dry weight) in each sediment layer (Top, Middle, and Bottom) per year. Each concentration is represented by the mean ± 95% confidence intervals.

| **Year** | **Layer** | **By season** | | **By location** | | **By cell** | |
| --- | --- | --- | --- | --- | --- | --- | --- |
|  |  | **Warm** | **Cool** | **Inflow** | **Outflow** | **1** | **2** |
| 2007 | Top | 12.2±14.2 | NA | 9.9±76.2 | 14.61±80.1 | 15.3±61.0 | 9.2±57.2 |
|  | Middle | 13.5±7.3 | NA | 13.3±22.9 | 13.8±67.3 | 15.3±28.0 | 11.8±16.5 |
|  | Bottom | 13.3±5.0 | NA | 14.0±7.6 | 12.3±36.9 | 14.0±7.6 | 12.3±36.9 |
| 2008 | Top | 28.5±34.2 | 175.0±526.8 | 131.4±582.7 | 72.2±246.1 | 126.6±593.3 | 76.9±234.7 |
|  | Middle | 17.5±6.9 | 9.5±4.1 | 12.7±13.5 | 13.3±13.3 | 12.9±11.7 | 13.3±16.4 |
|  | Bottom | 12.1±8.4 | NA | 12.0±21.5 | 12.2±12.7 | 12.8±13.7 | 11.3±19.6 |
| 2009 | Top | 337.1±482.2 | 38.7±76.6 | 196.4±457.5 | 175.1±684.2 | 146.3±675.7 | 215.6±450.7 |
|  | Middle | 11.2±10.4 | 10.5±6.2 | 10.9±7.5 | 10.7±8.4 | 11.2±7.6 | 10.5±7.8 |
|  | Bottom | 10.8±3.1 | NA | 10.9±3.3 | 10.6±9.3 | 10.9±5.3 | 10.7±6.1 |
| 2010 | Top | 326.4±647.8 | 259.9±622.0 | 495.7±737.5 | 90.6±87.0 | 252.7±612.2 | 333.5±654.5 |
|  | Middle | 14.7±13.1 | 10.9±11.4 | 14.7±5.1 | 10.9±16.6 | 12.9±11.4 | 12.6±11.5 |
|  | Bottom | 10.7±5.0 | 7.4±4.4 | 8.6±5.6 | 10.3±5.7 | 9.1±6.4 | 9.8±5.2 |
| 2011 | Top | 207.4±313.0 | 341.1±297.9 | 285.8±333.2 | 62.6±56.6 | 193.8±279.9 | 154.7±336.1 |
|  | Middle | 15.6±12.9 | 11.1±11.8 | 14.8±12.7 | 11.9±12.8 | 13.9±11.9 | 12.7±13.9 |
|  | Bottom | 8.0±2.0 | 5.6±3.9 | 5.8±3.6 | 7.8±3.3 | 7.2±4.5 | 6.4±3.4 |
| 2012 | Top | 367.7±439.5 | 371.7±583.3 | 535.0±577.1 | 204.4±160.5 | 380.6±452.9 | 358.8±572.3 |
|  | Middle | 12.6±16.3 | 13.3±8.6 | 17.1±18.7 | 10.4±5.1 | 14.2±11.6 | 11.6±12.4 |
|  | Bottom | 6.4±3.2 | 7.8±5.6 | 7.3±6.5 | 6.8±1.8 | 7.5±5.0 | 6.5±3.6 |
| 2013 | Top | 293.0±290.3 | 356.8±398.2 | 361.9±336.2 | 279.3±339.3 | 361.8±345.3 | 288.7±334.3 |
|  | Middle | 11.9±5.2 | 26.0±44.7 | 28.2±54.1 | 12.16±3.1 | 17.1±22.2 | 26.4±94.5 |
|  | Bottom | NA | 12.2±6.1 | 12.1±9.7 | 12.3±67.3 | 13.0±15.0 | 11.3±15.4 |

**References**

1. Xu, X.Y. and G.L. Mills, *Do constructed wetlands remove metals or increase metal bioavailability?* Journal of Environmental Management, 2018. **218**: p. 245-255.
