# Supplementary material for: Temporal deposition of copper and zinc in the sediments of metal removal constructed wetlands
Source: PLoS One. 2021 Aug 3;16(8):e0255527. doi: 10.1371/journal.pone.0255527 (PMC8330884; doi:10.1371/journal.pone.0255527)
Supplement: S1 Text — (DOCX) [file pone.0255527.s014.docx]

**Text S1**

**Model Selection**

Flowchart (Fig S2) describes the model selection process which involved including all possible explanatory variables (year, season, log_10_TC, log_10_TN) in the linear model (lm). Second, multiple collinearities were tested among the fixed effects by measuring Pearson’s correlation coefficients, and by testing the variance inflation factor using the “*vif*” function from the “car” package. Pearson’s correlation coefficient of 0.93 and a high *vif* value (> 17) indicated high collinearity between TC and TN. Therefore, we only kept log_10_TC in the model (Fig. S3, Table S1). Third, the evaluation of residual homogeneity was performed by plotting fitted vs standardized residuals. When a clear pattern in the residuals was encountered, several variance covariate structures were tested to minimize the residual heterogeneity. Fourth, the models with variance covariance structures that produced homogenous residuals were refitted with the generalized least squares using the restricted maximum likelihood estimation (*REML*). All generated models were inspected for residual homogeneity and were also compared to one another based on the Akaike’s information criterion (*AIC*). The model with the lowest AIC was used in the following steps. Fifth, the optimum fixed structure was determined using the backward selection process to eliminate the least statistically significant model term starting from higher to lower level interactions. Sixth, the resulting model was refitted excluding the least significant terms using the maximum likelihood ratio test. Finally, model validation was carried out in three steps; first, by evaluating the homogeneity of residuals using graphic tools and the Bartlett’s test of homogeneity. Second, by checking the normality graphically and by using the Shapiro-Wilk normality test. Finally, the independence of residuals was evaluated by checking for potential patterns when Pearson’s normalized residuals were plotted against all main effects. A significance level of α at 0.05 was used throughout the analysis.
