# Supplementary material for: Temporal deposition of copper and zinc in the sediments of metal removal constructed wetlands
Source: PLoS One. 2021 Aug 3;16(8):e0255527. doi: 10.1371/journal.pone.0255527 (PMC8330884; doi:10.1371/journal.pone.0255527)
Supplement: Attachment — Submitted filename: PONE-D-21-10299_reviewer_Comments.pdf [file pone.0255527.s022.pdf]

This is an original research paper on developing a pattern evaluation of heavy metals (Cu, Zn) settlement in the surface constructed wetland by time and Total Carbon (TC). The topic selected by the authors is appreciated in the specific domain of science and engineering for several purposes like contamination treatment at a lower price and lower environmental impact concern. Nonetheless, the work needs to be improved to reach the level of publication for readers of the respected PLOS ONE journal.

Abstract:

1. This needs precise information on how the outcomes of the linear regression model are going to be impacted.
2. Cu and Zn changed the value reported so TC needs to report as well.
3. Line 40: …On the other hand, using the generalized least squares extension with the linear regression model allowed for unequal variance, and thus was more successful in explaining Zn deposition pattern… what does mean 'more successful'? rewrite it.

Introduction:

4. Line50: … relatively cheaper… how? Explain it in detail.
5. The review assessment has been poorly drafted. Add more recent (5 years) papers.
   https://link.springer.com/article/10.1007/s11356-020-11775-z
   https://www.sciencedirect.com/science/article/abs/pii/S0048969721001388
6. There is no text belong why the authors used generalized generalized least squares extension with the linear regression model while there are more advanced approaches available?

Methods:

7. Need more explanation in Study site: write all the tributary or contribution to the site.
8. Why authors used limited no. of influencing parameters? Is there any problem to get more no. of those parameters metrological and/or climatological?
9. Write all functions of the code used in the 'Italic' font.

Results and Discussion:

10. Need statistical examination such as min, max, sd, etc in Table 1
11. FigureS1: replace with more presentable with all needed information
12. Better draw PCA Biplot to analyzed the dim strength between the factors.
13. Add regression equation in all scatter plots for showing the correlation mathematically how stronger?
14. Table S2: better add into the respective graph
15. In discussion: add how this changed value (within the used years) have an impact on the environment and local community and what measure should take to mitigate it for example several adsorption studies have been applied:
    https://www.sciencedirect.com/science/article/abs/pii/S0045653521006317
    https://link.springer.com/article/10.1007/s11356-021-12836-7

Conclusion:

16. Add the weakness of the study.

17. Add future objective of the research including the applying machine learning approach to predict the sediment for example
    https://www.sciencedirect.com/science/article/abs/pii/S0304389420314783
    https://www.sciencedirect.com/science/article/abs/pii/S026974912036351X
