# Supplementary material for: Temporal deposition of copper and zinc in the sediments of metal removal constructed wetlands
Source: PLoS One. 2021 Aug 3;16(8):e0255527. doi: 10.1371/journal.pone.0255527 (PMC8330884; doi:10.1371/journal.pone.0255527)
Supplement: Attachment — Submitted filename: PLOS ONE.docx [file pone.0255527.s020.docx]

**COMMENTS FOR THE AUTHORS:**

Overall, the calibration and validation processes are acceptable. So, I found the manuscript in a favorable format to be published on PLOS ONE journal with the topic “Temporal Deposition of Copper (Cu) and Zinc (Zn) in the Sediments of Metal Removal Constructed Wetlands”. This research project has the scientific background enough to be published, and the authors present a fair amount of data and a reasonable list of references. However, there is some comments needed to be observing.

1. Avoid using the first person pronouns "I or we” in your writing, and the most common reason given for this is that readers may regard such writing as being subjective, whereas science is all about objectivity.
2. manuscript should have some novelty in its work
3. remove “map” from the title of Fig S1 and line 101
4. fig S1 need to add the size and the scale
5. My suggestion to add Figure of study area to the manuscript
6. the methods section very long , please minimize it
7. the conclusion section very short and need more info, you need make balance between sections
8. Fig S5, S7, S8 and S9 titles need modify according to the charts type.
