# Supplementary material for: Temporal deposition of copper and zinc in the sediments of metal removal constructed wetlands
Source: PLoS One. 2021 Aug 3;16(8):e0255527. doi: 10.1371/journal.pone.0255527 (PMC8330884; doi:10.1371/journal.pone.0255527)
Supplement: Attachment — Submitted filename: Reviwe Temporal Deposition of Copper (Cu) and Zinc (Zn) in the Sediments of Metal Removal.docx [file pone.0255527.s021.docx]

**Review**

Temporal Deposition of Copper (Cu) and Zinc (Zn) in the Sediments of Metal Removal Constructed Wetlands

I consider this paper very good and is an important addition to the literature , this research predicted Copper (Cu) and Zinc (Zn) deposition patterns , the research examined and studied the effects of time, seasons, and total carbon (TC) on Copper (Cu) and Zinc (Zn) deposition in the surface sediments of the H-02 constructed wetland on the Savannah River Site .

This research Covering both warm (April-September) and cool (October-March) seasons, several sediment cores were collected twice a year from the constructed wetland cells over 7 years, from 2007 to 2013.

This research used the Generalized Least Squares (GLS) & Linear Regression Model (LRM), linear regression model explained the behaviour and the variability of Cu deposition in the sediments , using the (GLS) extension with the (LRM)allowed for unequal variance .

Here are several comments :

1- Interesting and within the journal scope manuscript and it is properly organized .
2- Have appropriate forms and have academic value.
3- Methodology flowchart for the modelling procedure is needed.
4- The data interpreted and analysed statistically in appropriate manner.
5- Presents results in a clear way .
6- The conclusions reached are properly validated by the  results.
7- The paper (Approved) ,can accepted for publication with minor revisions .
