# Supplementary material for: Temporal deposition of copper and zinc in the sediments of metal removal constructed wetlands
Source: PLoS One. 2021 Aug 3;16(8):e0255527. doi: 10.1371/journal.pone.0255527 (PMC8330884; doi:10.1371/journal.pone.0255527)
Supplement: Attachment — Submitted filename: Response to Reviewers.docx [file pone.0255527.s023.docx]

**Response to Reviewers**

**Disclaimer:** Please don’t mind the inconsistencies in line numbers on the track changes-version, this is a common issue in word

Reviewer 1

I consider this paper very good and is an important addition to the literature , this research predicted Copper (Cu) and Zinc (Zn) deposition patterns , the research examined and studied the effects of time, seasons, and total carbon (TC) on Copper (Cu) and Zinc (Zn) deposition in the surface sediments of the H-02 constructed wetland on the Savannah River Site .

This research Covering both warm (April-September) and cool (October-March) seasons, several sediment cores were collected twice a year from the constructed wetland cells over 7 years, from 2007 to 2013.

This research used the Generalized Least Squares (GLS) & Linear Regression Model (LRM), linear regression model explained the behaviour and the variability of Cu deposition in the sediments , using the (GLS) extension with the (LRM)allowed for unequal variance .

Here are several comments :

1- **Interesting and within the journal scope manuscript and it is properly organized .**
2- **Have appropriate forms and have academic value.**
3- **Methodology flowchart for the modelling procedure is needed.**

We have created a flow-chart, please refer to figure S2 in the SI file

4- **The data interpreted and analysed statistically in appropriate manner.**
5- **Presents results in a clear way .**
6- **The conclusions reached are properly validated by the  results.**
7- **The paper (Approved) ,can accepted for publication with minor revisions .**

We thank the reviewer for their comments.

Reviewer 2

I read the manuscript and found it very good, as it represents a scientific research of original data with an applied character that simulates the environmental treatment of heavy elements (Cu and Zn). Field experiments, statistics and chemical analyses have been performed in high technical standard and results were presented in a clear manner mostly except for some places of the manuscript. The manuscript meets all applicable standards for the ethics of experimentation and research integrity. However, I recommend to accept the manuscript after major revision and the following points should be addressed:

1. **Title must be concise and informative, so the abbreviation Cu and Zn should be deleted and title become “Temporal Deposition of Copper and Zinc in the Sediments of Metal Removal Constructed Wetlands”.**

We appreciate the reviewer’s comment. However, keeping the elemental formula for both metals improves the searchability and visibility of our article to other researchers.

2. **Line 59: The expression “media/soils” doesn’t be accepted, please change it in another style.**

We fixed this issue, please refer to line 67.

3. **Line 103-104: The author stated “Briefly, the Tritium facility discharges wastewater through source pipes into a rectangular pool where water is retained to provide hydrological control”. How much is the tritium wastewater discharged through source pipes into a rectangular basin?**

We thank the reviewer for this comment. We don’t have access to such data.

4. **Line 123-124: Surface standing water was carefully siphoned off from the cores avoiding disturbing the flocculent layer. To what extent surface standing water can be siphoned?**

Ideally, we were careful not to disturb/siphon the flocculant layer (soft organic rich floccule-like material). The amount of surface standing water in sediment core was variable depending on the water level in the wetland at the time of sampling.

5. **Line 129-130: Dried sediments were passed through a 2 mm sieve. I think after sediments be dried will need gentle grinding to be sieved easily.** The sediments were only sieved, no grinding was applied**. Line 176-177: In the model selection process, we followed the protocol mentioned in Zuur et al. 2009, (Zuur et al. 177 2009). Please, it is better is not to repeat the reference “Zuur et al. 2009” twice, try to reformulate the statement. Line 181: Please do not use do not use the active voice and the pronoun “we” as mentioned in “Therefore, we only kept log10TC in the model”.by the same way, look at Line 184; “Fourth, we refitted the model with the generalized”; Line 188; “Fifth, we specified the optimum fixed structure using the backward selection”; Lines191 and 192 “first, we evaluated the homogeneity of residuals using graphic tools and the Bartlett’s test of homogeneity. Second, we checked the normality graphically and by using the….”; and lines 194-195 “We used α at 0.05 as the significance level”.**

We have fixed all these issues, please refer to lines 192-197 in the manuscript and Text S1 in the SI file.

6. **Line 198: I suggest to change that title to “Deposition of Cu and Zn on the interface water-sediments”.**

We appreciate the reviewer comment, but we analysed the metals in the whole sediment cores and not just the very top part in contact with the standing water above.

7. **In both models, we find ambiguity in which layers the copper and zinc are deposited more? and why?**

Only surface sediments metal data were used in the model. We have mentioned the rationale behind this in the text (Lines 176-179), but briefly, the surface sediments have considerably higher Cu, Zn, and carbon content than the layers underneath.

8. **Please explain the role of Total carbon in the absorption process and its absorption capacity.**

We have not performed adsorption isotherm experiments that would help figure out the adsorption process or capacity. While this would be an interesting aspect, it was beyond the scope of the current study.

9. **The best was to analyze the minerals and diagnose the type of clay minerals. As is known, every clay mineral has specific value of cation exchange capacity.**

We thank and agree with the reviewer. However, we have not performed such analysis.

10. **It is better to make mineralogy study for the soil sample.**

We appreciate the reviewer comment, this would be an interesting analysis for another follow up study. In this work, we were particularly interested in heavy metal accumulation trend over the years and seasons.

11. **Line 351 “Heavy metal (Cu and Zn)”, I think lead and zinc represent a plural state, not singular, so it is better to write the term heavy metals, not heavy metal.**

We appreciate the reviewer comment, please refer to line 540.

12. **If you use British language, the Acknowledgments” write as “Acknowledgements”.**

We appreciate the reviewer comment, please refer to line 556.

Reviewer 3

Overall, the calibration and validation processes are acceptable. So, I found the manuscript in a favorable format to be published on PLOS ONE journal with the topic “Temporal Deposition of Copper (Cu) and Zinc (Zn) in the Sediments of Metal Removal Constructed Wetlands”. This research project has the scientific background enough to be published, and the authors present a fair amount of data and a reasonable list of references. However, there is some comments needed to be observing.

1. **Avoid using the first person pronouns "I or we” in your writing, and the most common reason given for this is that readers may regard such writing as being subjective, whereas science is all about objectivity.**

We have fixed all these issues, please refer to lines 192-197 in the manuscript and Text S1 in the SI file.

2. **manuscript should have some novelty in its work**

We thank the reviewer for the feedback. We think that this works novelty comes from our use of statistical tools and 7 years- worth of data to give us insights about the accumulation pattern of heavy metals in the surface sediments of the H-02 wetland. We believe that this is an invaluable tool which we could build upon for the next years to predict the performance and thus the efficiency of the H-02 wetland, and to protect the environment and the wildlife at the Savannah River site.

3. **remove “map” from the title of Fig S1 and line 101**

We have replaced the word “map” with: Schematic diagram”, line 112, please refer to Fig S1 title (line 570), and Fig S1 title in the SI Paper-Track changes file.

4. **fig S1 need to add the size and the scale**

We have updated the figure with the required information

5. **My suggestion to add Figure of study area to the manuscript**

We have already provided a schematic diagram of the study area (please see Fig S1 in the SI file). Also, we don’t have access to an airborne image of the study area.

6. **the methods section very long , please minimize it**

We fixed this issue by moving model selection details to the SI file, please refer to Text S1 in the SI file and lines 192-197 in the manuscript.

7. **the conclusion section very short and need more info, you need make balance between sections**

We fixed this issue, please refer to lines 540-554.

8. **Fig S5, S7, S8 and S9 titles need modify according to the charts type.**

We have modified the titles, please notice that the figure numbers have been updated, please see Fig S6, S8, S9, and S10.

Reviewer 4

This is an original research paper on developing a pattern evaluation of heavy metals (Cu, Zn)

settlement in the surface constructed wetland by time and Total Carbon (TC). The topic

selected by the authors is appreciated in the specific domain of science and engineering for

several purposes like contamination treatment at a lower price and lower environmental impact

concern. Nonetheless, the work needs to be improved to reach the level of publication for

readers of the respected PLOS ONE journal.

Abstract:

**1. This needs precise information on how the outcomes of the linear regression model are**

**going to be impacted.**

We believe that we have provided precise and summarized details of the linear regression model in the abstract (lines 40-45), we would appreciate it if the reviewer would give us more clarification of what exactly we should be adding in the abstract.

**2. Cu and Zn changed the value reported so TC needs to report as well.**

We thank the reviewer for this comment. But since we were more focused on Cu and Zn accumulation, we dropped the TC from line 37.

**3. Line 40: …On the other hand, using the generalized least squares extension with the**

**linear regression model allowed for unequal variance, and thus was more successful in**

**explaining Zn deposition pattern… what does mean ‘more successful’? rewrite it.**

We added a clarifying sentence, please refer to lines 41-43.

Introduction:

**4. Line50: … relatively cheaper… how? Explain it in detail.**

We added a sentence to clarify our point, please refer to lines 50-51.

**5. The review assessment has been poorly drafted. Add more recent (5 years) papers.**

We added more recent references, please check lines 52, 54, 68-69, 77, 81, 82, 84

https://link.springer.com/article/10.1007/s11356-020-11775-z

https://www.sciencedirect.com/science/article/abs/pii/S0048969721001388

**6. There is no text belong why the authors used generalized generalized least squares**

**extension with the linear regression model while there are more advanced approaches**

**available?**

The generalized least squares (GLS) extension is used to handle residual heterogeneity. We added this clarification in line 115.

Methods:

**7. Need more explanation in Study site: write all the tributary or contribution to the site.**

There is no tributary or contribution to the site

**8. Why authors used limited no. of influencing parameters? Is there any problem to get**

**more no. of those parameters metrological and/or climatological?**

We thank the reviewer for their comment. We have not measured these parameters.

**9. Write all functions of the code used in the ‘Italic’ font.**

We have made the required changes. Please refer to Text S1 in the SI file.

Results and Discussion:

10. **Need statistical examination such as min, max, sd, etc in Table 1**

We have added the maximum, minimum, and replaced 95% confidence intervals with the standard deviation. Please refer to table 1 in the manuscript as well as lines 210 and 214.

11. **FigureS1: replace with more presentable with all needed information**

We have added the required information, please see Fig. S1 in the SI file.

12. **Better draw PCA Biplot to analyzed the dim strength between the factors.**

We appreciate the reviewer comment. However, in this work, we were more interested in addressing the accumulation behaviour of the metals in the wetland surface sediment over time and seasons. We will definitely consider PCA as a valuable tool in our upcoming work.

**13. Add regression equation in all scatter plots for showing the correlation mathematically**

**how stronger?**

We removed table S2 and added regression equations and correlation coefficients to the scatter plots. Please refer to “Fig3NEW” in the attachments.

**14.Table S2: better add into the respective graph**

Please see our response to comment 13 above.

**15. In discussion: add how this changed value (within the used years) have an impact on**

**the environment and local community and what measure should take to mitigate it for**

**example several adsorption studies have been applied:**

**https://www.sciencedirect.com/science/article/abs/pii/S0045653521006317**

**https://link.springer.com/article/10.1007/s11356-021-12836-7**

We thank the reviewer for this comment, but we have not performed any studies that would answer this question.

**Conclusion:**

**16. Add the weakness of the study.**

We have added weaknesses and future directions in the conclusion section (lines 548-562)

**17. Add future objective of the research including the applying machine learning approach to predict the sediment for example**

**https://www.sciencedirect.com/science/article/abs/pii/S0304389420314783**

**https://www.sciencedirect.com/science/article/abs/pii/S026974912036351X**

We have added a sentence that addressed this suggestion, please refer to lines 557-559
