# Supplementary material for: Temporal deposition of copper and zinc in the sediments of metal removal constructed wetlands
Source: PLoS One. 2021 Aug 3;16(8):e0255527. doi: 10.1371/journal.pone.0255527 (PMC8330884; doi:10.1371/journal.pone.0255527)
Supplement: Attachment — Submitted filename: Review report.docx [file pone.0255527.s024.docx]

**Review report (Round 2**)

I have reviewed the required amendments and found a good response from the researcher. He accomplished most of the basic points and answered some of them adequately. I therefore find the manuscript now is more quality, so I recommend to give an accept decision.

Additionally, I advise the author that I prefer to remove the chemical element symbols (Cu and Zn) from the manuscript title. It doesn’t mean thing to be included in the title.
