# Supplementary material for: Temporal deposition of copper and zinc in the sediments of metal removal constructed wetlands
Source: PLoS One. 2021 Aug 3;16(8):e0255527. doi: 10.1371/journal.pone.0255527 (PMC8330884; doi:10.1371/journal.pone.0255527)
Supplement: Attachment — Submitted filename: Reviewer comment.docx [file pone.0255527.s019.docx]

**Reviewer comments**

**Title: Temporal Deposition of Copper (Cu) and Zinc (Zn) in the Sediments of Metal Removal Constructed Wetlands**

I read the manuscript and found it very good, as it represents a scientific research of original data with an applied character that simulates the environmental treatment of heavy elements (Cu and Zn). Field experiments, statistics and chemical analyses have been performed in high technical standard and results were presented in a clear manner mostly except for some places of the manuscript. The manuscript meets all applicable standards for the ethics of experimentation and research integrity. However, I recommend to accept the manuscript after major revision and the following points should be addressed:

1. Title must be concise and informative, so the abbreviation Cu and Zn should be deleted and title become “Temporal Deposition of Copper and Zinc in the Sediments of Metal Removal Constructed Wetlands”.
2. Line 59: The expression “media/soils” doesn’t be accepted, please change it in another style.
3. Line 103-104: The author stated “Briefly, the Tritium facility discharges wastewater through source pipes into a rectangular pool where water is retained to provide hydrological control”. How much is the tritium wastewater discharged through source pipes into a rectangular basin?
4. Line 123-124: Surface standing water was carefully siphoned off from the cores avoiding disturbing the flocculent layer. To what extent surface standing water can be siphoned?
5. Line 129-130: Dried sediments were passed through a 2 mm sieve. I think after sediments be dried will need gentle grinding to be sieved easily. Line 176-177: In the model selection process, we followed the protocol mentioned in Zuur et al. 2009, (Zuur et al. 177 2009). Please, it is better is not to repeat the reference “Zuur et al. 2009” twice, try to reformulate the statement. Line 181: Please do not use do not use the active voice and the pronoun “we” as mentioned in “Therefore, we only kept log10TC in the model”.by the same way, look at Line 184; “Fourth, we refitted the model with the generalized”; Line 188; “Fifth, we specified the optimum fixed structure using the backward selection”; Lines191 and 192 “first, we evaluated the homogeneity of residuals using graphic tools and the Bartlett’s test of homogeneity. Second, we checked the normality graphically and by using the….”; and lines 194-195 “We used α at 0.05 as the significance level”.
6. Line 198: I suggest to change that title to “Deposition of Cu and Zn on the interface water-sediments”.
7. In both models, we find ambiguity in which layers the copper and zinc are deposited more? and why?
8. Please explain the role of Total carbon in the absorption process and its absorption capacity.
9. The best was to analyze the minerals and diagnose the type of clay minerals. As is known, every clay mineral has specific value of cation exchange capacity.
10. It is better to make mineralogy study for the soil sample.
11. Line 351 “Heavy metal (Cu and Zn)”, I think lead and zinc represent a plural state, not singular, so it is better to write the term heavy metals, not heavy metal.
12. If you use British language, the Acknowledgments” write as “Acknowledgements”.

Salih M Awadh

salih.awad@sc.uobaghdad.edu.iq
